# Xenoestrogens at Picomolar to Nanomolar Concentrations Trigger Membrane Estrogen Receptor-α–Mediated Ca^2+^ Fluxes and Prolactin Release in GH3/B6 Pituitary Tumor Cells

**DOI:** 10.1289/ehp.7505

**Published:** 2005-01-14

**Authors:** Ann L. Wozniak, Nataliya N. Bulayeva, Cheryl S. Watson

**Affiliations:** Department of Human Biological Chemistry and Genetics, University of Texas Medical Branch, Galveston, Texas, USA

**Keywords:** bisphenol A, coumestrol, DDE, DES, diethylstilbestrol, dieldrin, endosulfan, estrogen receptor-α, exocytosis, L-type channels, membrane, nonylphenol, phytoestrogen, prolactin, xenoestrogen

## Abstract

Xenoestrogens (XEs) are widespread in our environment and are known to have deleterious effects in animal (and perhaps human) populations. Acting as inappropriate estrogens, XEs are thought to interfere with endogenous estrogens such as estradiol (E_2_) to disrupt normal estrogenic signaling. We investigated the effects of E_2_ versus several XEs representing organochlorine pesticides (dieldrin, endosulfan, *o*′*p*′-dichlorodiphenylethylene), plastics manufacturing by-products/detergents (nonylphenol, bisphenol A), a phytoestrogen (coumestrol), and a synthetic estrogen (diethylstilbestrol) on the pituitary tumor cell subline GH3/B6/F10, previously selected for expression of high levels of membrane estrogen receptor-α. Picomolar to nanomolar concentrations of both E_2_ and XEs caused intracellular Ca^2+^ changes within 30 sec of administration. Each XE produced a unique temporal pattern of Ca^2+^ elevation. Removing Ca^2+^ from the extracellular solution abolished both spontaneous and XE-induced intracellular Ca^2+^ changes, as did 10 μM nifedipine. This suggests that XEs mediate their actions via voltage-dependent L-type Ca^2+^ channels in the plasma membrane. None of the Ca^2+^ fluxes came from intracellular Ca^2+^ stores. E_2_ and each XE also caused unique time- and concentration-dependent patterns of prolactin (PRL) secretion that were largely complete within 3 min of administration. PRL secretion was also blocked by nifedipine, demonstrating a correlation between Ca^2+^ influx and PRL secretion. These data indicate that at very low concentrations, XEs mediate membrane-initiated intracellular Ca^2+^ increases resulting in PRL secretion via a mechanism similar to that for E_2_, but with distinct patterns and potencies that could explain their abilities to disrupt endocrine functions.

Environmental chemicals with estrogenic activities [xenoestrogens (XEs)] have been implicated in harmful endocrine effects on animals and humans such as the feminization of male animal populations (Kloas et al. 1999; Sumpter 1995), reproductive tract malformations and endometriosis (Gotz et al. 2001; Lee 1998; Steinmetz et al. 1998), disorganization of the central nervous system (Laessig et al. 1999; Oka et al. 2003), and breast and ovarian cancer (Brown and Lamartiniere 1995; Mathur et al. 2002). By acting as estrogen mimetics and binding to estrogen receptors (ERs), XEs may disrupt normal endocrine function, leading to reproductive failure and the induction of tumors in estrogen-sensitive tissues. XEs can also cause alteration of hormone levels via changes in hormone production, metabolism, or transport (Sonnenschein and Soto 1998).

There are many potential endocrine-disrupting chemicals that are prevalent in the environment, or to which humans have been otherwise exposed (Singleton and Khan 2003); in this study we examined several representative compounds. Erroneously used to prevent miscarriages in the 1950s and 1960s, diethylstilbestrol (DES) acts developmentally as a potent estrogen agonist, causing adenocarcinomas, squamous neoplasia of the vagina and cervix (Hatch et al. 2001), oligospermia (vom Saal et al. 1997), and infertility (Palmer et al. 2001). The pesticide *o*′*,p*′-dichlorodiphenylethylene (DDE) and its metabolites can disorder prostate maturation (Gray et al. 1999). Endocrine disruptors are known to have great impact during fetal development when endogenous hormones regulate cell differentiation and growth, and thus slight alterations in hormonal activity due to endocrine disruption can lead to irreversible changes (Derfoul et al. 2003). However, the abilities of XEs to disrupt adult endocrine function and perhaps to exacerbate estrogen-dependent tumor growth (Soto et al. 1995) are also of concern. We also examined other XEs reported to have estrogen-like activities: detergents such as nonylphenol and bisphenol A (BPA), the organochlorine pesticides dieldrin and endosulfan, and the phytoestrogen coumestrol.

Estrogenic actions have been well studied with respect to genomic responses mediated by nuclear ERs. The nuclear ER-mediated gene transcription responses to XEs are very weak [effective only at 1,000- to 10,000-fold higher concentrations than estradiol (E_2_; Massaad and Barouki 1999; Stevens et al. 1994; Witorsch 2002)], leading some to suggest that their presence in our environment is relatively harmless. However, in addition to classical genomic actions, estrogens can act through non-genomic or membrane-initiated signaling pathways via a membrane form of ER (mER). Examples of such actions are alterations in G-protein–coupled receptor responses, protein phosphorylation, lysosomal membrane destabilization, K^+^ and Ca^2+^ channel activation, and nitric oxide secretion (reviewed by Watson and Gametchu 1999, 2003). XE actions via nongenomic pathways remain largely unstudied.

Ca^2+^ responses to extracellular stimuli can lead to changes in cell motility, intra- and extracellular signaling processes, and rapid hormone secretion [including prolactin (PRL)] through exocytosis (Campbell 1990; Pappas et al. 1994; Watson et al. 1999a). Changes in PRL secretion are associated with hormonal regulation of lactation, cell proliferation, the cellular immune response, and parental/maternal behavior (Freeman et al. 2000). We recently showed that picomolar to nanomolar concentrations of E_2_ and XEs can initiate mitogen-activated protein kinase activation and that several signaling pathways, including Ca^2+^ elevation, may participate in this kinase activation (Bulayeva et al. 2004; Bulayeva and Watson 2004). We also demonstrated the ability of a physiological estrogen (E_2_) to elicit cellular Ca^2+^ influx via a membrane version of ER-α(Bulayeva et al. 2005). Here we investigate in more detail the ability of several XEs (DES, coumestrol, *p*-nonylphenol, BPA, DDE, dieldrin, and endosulfan) to induce rapid intracellular Ca^2+^ changes leading to PRL secretion in mER-α–enriched or depleted sublines of GH3/B6 cells (Pappas et al. 1994). Mis-regulation of such cellular signaling events by XEs could lead to damaging endocrine disruptions such as tissue malformation, cancer, and reproductive system malfunctions.

## Materials and Methods

We purchased phenol red-free Dulbecco modified Eagle medium (DMEM) from Mediatech (Herndon, VA); horse serum from Gibco BRL (Grand Island, NY); defined supplemented calf sera and fetal bovine sera from Hyclone (Logan, UT); endosulfan and DDE from Ultra Scientific (North Kingstown, RI); and all other XEs from Sigma (St. Louis, MO). Paraformaldehyde and glutaraldehyde were purchased from Fisher Scientific (Pittsburgh, PA). We purchased nifedipine and thapsigargin from Calbiochem (San Diego, CA) and Fura-2/AM from Molecular Probes (Eugene, OR). All other materials were purchased from Sigma.

### Cell culture.

Clonal rat prolactinoma cell lines were selected for high (GH3/B6/F10) and low (GH3/B6/D9) expression of mER-α (Pappas et al. 1994). For the present experiments, cells were subsequently reselected by immunopanning for highly enriched and deficient expression of mER-α and then used between passages 2 and 12. Cells were routinely cultured in DMEM containing 12.5% horse serum, 2.5% defined supplemented calf serum, and 1.5% fetal calf serum. For individual experiments, cells were deprived of steroids for 48 hr after plating by replacing serum-containing DMEM with DMEM containing 5 μg/mL insulin-transferrin and 5 ng/mL sodium selenite, 0.1% bovine serum albumin (BSA), 20 mM sodium pyruvate, and 25 mM HEPES (DMEM/ITS). Immediately before the experiments, cells were incubated in DMEM alone for 1 hr.

### Ca^2+^ measurements.

GH3/B6 cell sublines were plated on poly-d-lysine–coated coverslips in wells of a six-well plate (10^5^ cells/well). After serum deprivation in DMEM/ITS and then DMEM, the cells were washed in Ringer’s solution (120 mM NaCl, 1.25 mM CaCl_2_, 4.7 mM KCl, 1.2 mM MgCl_2_, 20 mM HEPES, 10 mM glucose, 0.1% BSA; pH 7.4), loaded with 2 μM Fura-2/AM diluted in Ringer’s, wrapped in aluminum foil, and incubated at room temperature (RT) for 1 hr. The cells were washed twice and left to equilibrate in Ringer’s for 20 min at RT before imaging. E_2_ and XEs were administered using a perfusion pump system at a rate of 2 mL/min. Although responses to E_2_ continue during a 5-min hormonal treatment, these effects are reversible, taking about 5 min to wash out (Bulayeva et al. 2005). Imaging was performed using a TE200-IUC Quantitative Fluorescence Live-Cell and Multidimensional Imaging System equipped with a digital monochrome cooled CCD Roper Coolsnap HQ camera (Roper Scientific, Tucson, AZ). Ca^2+^ measurements were collected using the MetaFluor program (Universal Imaging, Downingtown, PA), making sure that only single cells were used as the region of interest. Data were recorded every second. Signals were obtained in dual excitation mode (340/380 nm), and the intracellular Ca^2+^ was calculated as a ratio (*R*_340/380_) of emission data collected at 510 nm after background subtraction. Intracellular Ca^2+^ was quantified by calculating the change in fluorescence ratio (*R* – *R*_0_) during a 5-min treatment period, normalized to the basal fluorescence value (*R*_0_) for each individual cell. These calculations for individual cells were then averaged to calculate the means and SEs for the population. Test and calibration solutions included Ca^2+^-free solution (Ringer’s without CaCl_2_ and with 2 mM EGTA), Ringer’s–20 mM KCl (Ringer’s with NaCl decreased to 105 mM and KCl increased to 20 mM), and maximum Ca^2+^ solution (Ringer’s with NaCl decreased to 112 mM and CaCl_2_ increased to 10 mM). KCl treatments were used at the end of each experiment to establish cell viability. Cells that did not respond transiently to KCl depolarization at the end of the experiment were eliminated from the composite calculations.

### PRL release and radioimmunoassay.

Cells (0.5–0.7 × 10^6^) were plated in poly-d-lysine–coated six-well plates. After serum deprivation in DMEM/ITS, this medium was removed and new DMEM/0.1% BSA with or without the appropriate reagent or vehicle control (ethanol) was added. The cells were incubated for 1, 3, 6, 10, or 15 min and centrifuged at 4°C, 350 × *g*, for 5 min. The supernatant was then collected and stored at −20°C until radioimmunoassay (RIA). Concentrations of PRL were determined using components of the rat PRL RIA kit from the National Institute of Diabetes and Digestive and Kidney Disease and the National Hormone and Pituitary Program (Baltimore, MD). Briefly, RIA buffer [80% phosphate-buffered saline (PBS), 20% DMEM, 2% normal rabbit serum], 100 μL cold standard (rat PRL-RP-3) or unknown sample, rPRL-s-9 antiserum (final dilution of 1:437,500 in RIA buffer), and [^125^I]-rat-PRL (PerkinElmer, Wellesley, MA, USA; using 15,000 counts per tube diluted in RIA buffer) were combined and incubated with shaking, overnight at 4°C. Anti-rabbit IgG (R-0881; Sigma) was added to a final dilution of 1:9, and the samples were incubated with shaking at RT for 2 hr. One milliliter of polyethylene glycol solution [1.2 M polyethylene glycol (P-6667; Sigma), 50 mM Tris, pH 8.6] was then added, and the samples were incubated with shaking at RT for 15 min. The samples were then centrifuged at 4,000 × *g* for 10 min at 4°C, the supernatant was decanted, and the pellet was counted in a Wizard 1470 Gamma Counter (PerkinElmer, Boston, MA). The PRL concentration was then calculated and normalized to the crystal violet values representing cell number.

### Crystal violet assay.

We used the crystal violet assay to determine cell number to which PRL concentrations were normalized. After collecting the supernatant from the PRL assay, cells were fixed by adding 1 mL fixative (2% paraformaldehyde, 0.1% glutaraldehyde, dissolved in PBS) per well. Sample plates were then rocked at RT for 30 min. The plates were then washed three times with deionized water and allowed to dry overnight. Crystal violet solution (1 mL of a 0.1% solution in water, filtered) was added to each well, and the plates were incubated with rocking at RT for 30 min. The plates were then washed and dried, and the dye was extracted with 1 mL per well of a 10% acetic acid solution (in water). After a 30 min incubation at RT, the absorbance at 590 nm was read in a model 1420 Wallac microplate reader (PerkinElmer, Boston, MA).

### Statistics.

We calculated the statistical significance of differences using Sigma Stat (version 3.0; Jandel Scientific, San Rafael, CA) and one-way analysis of variance.

## Results

### XEs increase intracellular Ca^2+^ levels.

**Figure 1:**
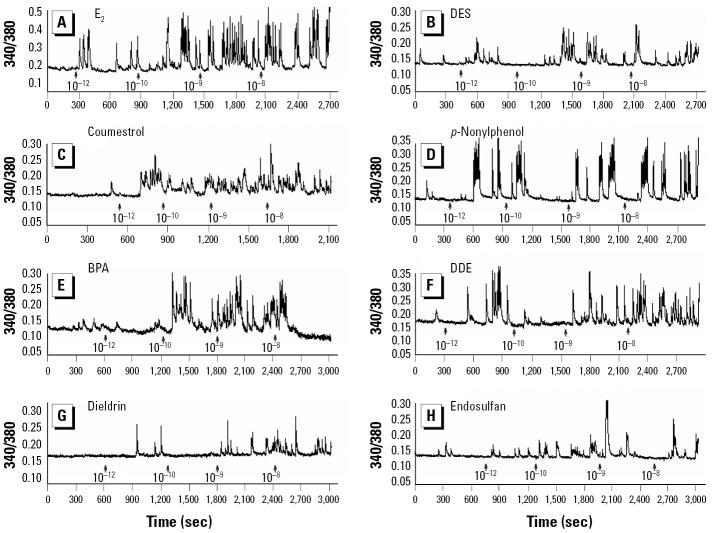
Intracellular Ca^2+^ changes induced by (*A*) E_2_, (*B*) DES, (*C*) coumestrol, (*D*) *p*-nonylphenol, (*E*) BPA, (*F*) DDE, (*G*) dieldrin, and (*H*) endosulfan. Each Ca^2+^ profile (340/380 nm trace) is a single representative cell. See “Materials and Methods” for details of experiments.

**Figure 2:**
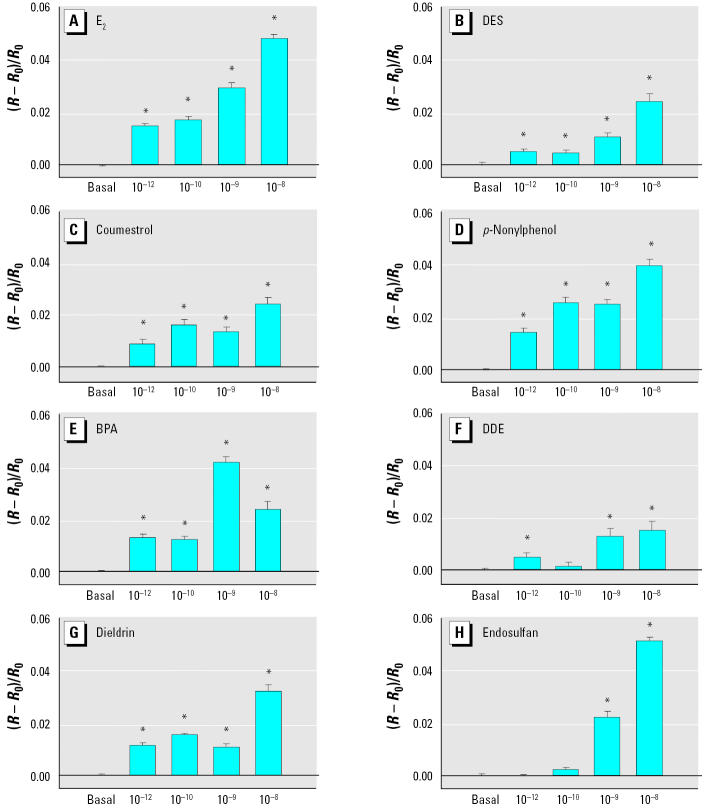
Intracellular Ca^2+^ changes induced by (*A*) E_2_ (*n* = 24 cells/3 experiments), (*B*) DES (*n* = 19 cells/3 experiments), (*C*) coumestrol (*n* = 12 cells /4 experiments), (*D*) *p*-nonylphenol (*n* = 12 cells/4 experiments), (*E*) BPA (*n* = 15 cells /4 experiments), (*F*) DDE (*n* = 7 cells /3 experiments), (*G*) dieldrin (*n* = 20 cells/3 experiments), and (*H*) endosulfan(*n* = 19 cells /4 experiments). Bars display the change in fluorescence ratio divided by the basal fluorescence [(*R* – *R*_0_)/*R*_0_], averaged from multiple imaged GH3/B6/F10 cells over several experiments (mean ± SE).
*Statistically significant from basal level (*p* < 0.05).

**Figure 3:**
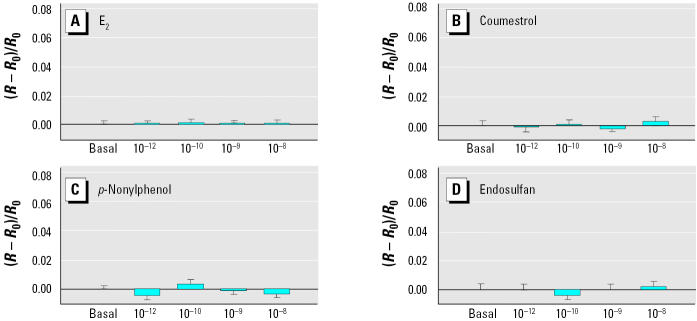
Lack of Ca^2+^ responses in mER-α–deficient D9 cells treated with (*A*) E_2_ (*n* = 28 cells/3 experiments), (*B*) coumestrol (*n* = 17 cells/3 experiments), (*C*) *p*-nonylphenol (*n* = 6 cells /3 experiments), and (*D*) endosul-fan (*n* = 7 cells/3 experiments). Bars display the change in fluorescence ratio divided by the basal fluorescence [(*R* – *R*_0_)/*R*_0_], averaged for multiple imaged cells over several experiments (mean ± SE). No values were significantly different from the basal.

We previously showed that E_2_ can trigger a rapid and reversible (within 5 min) intracellular Ca^2+^ change (increase from basal level) in our mER-α–enriched (F10) rat prolactinoma cell subline, whereas the mER-α–depleted (D9) subline showed no Ca^2+^ response (Bulayeva et al. 2005). In the present study, E_2_ and all XEs studied initiated a change in intracellular Ca^2+^ levels (increased frequency and/or amplitude) within 30 sec of administration in mER-α–enriched F10 cells (Figures 1 and 2). Untreated cells during this testing period do not show a response (Bulayeva et al. 2005). Each compound produced a unique dose–response pattern with respect to potency, peak height, and/or frequency. All XEs elicited concentration-dependent responses; at the highest concentrations tested (10^−9^–10^−8^ M), although all XEs caused a response, generally they did so less robustly or potently when compared with E_2_. E_2_ showed a significant intracellular Ca^2+^ change at concentrations as low as 10^−12^ M, and increased with concentration. DES gave a similar response, although somewhat less robustly. Coumestrol was also effective at all tested concentrations; however, its maximal response (10^−8^ M) was half that caused by E_2_. Nonylphenol elicited concentration-dependent increases in Ca^2+^ influxes with similar characteristics to E_2_, but with the most robust response at 10^−8^ M being slightly lower than that for E_2_. BPA displayed a maximal response at 10^−9^ M that declined at a higher concentration. DDE produced the smallest Ca^2+^ elevations. Dieldrin elicited a Ca^2+^ change at all concentrations. Endosulfan caused no intracellular Ca^2+^ changes at the lowest concentrations, yet 10^−9^ M and 10^−8^ M produced quite robust influx. When we examined individual cells, we found that they were heterogeneous in their responsiveness to XEs, as we have observed previously in responses to E_2_ (Watson et al. 1999a). Overall, 82% of the cells tested responded to treatment with E_2_ or XEs, but some cells did not respond at all. We did not average nonresponders into the composite measurements (Figure 2), and the error measurements shown represent cells with differing response capabilities. No Ca^2+^ changes occurred upon the administration of XEs of any concentration in the mER-α–deficient D9 cells (Figure 3); for these studies we tested only a single representative compound from each category (E_2_, phytoestrogen, detergent, and organochlorine pesticide).

### Intracellular Ca^2+^ stores are not responsible for E_2_-induced Ca^2+^ level changes.

**Figure 4:**
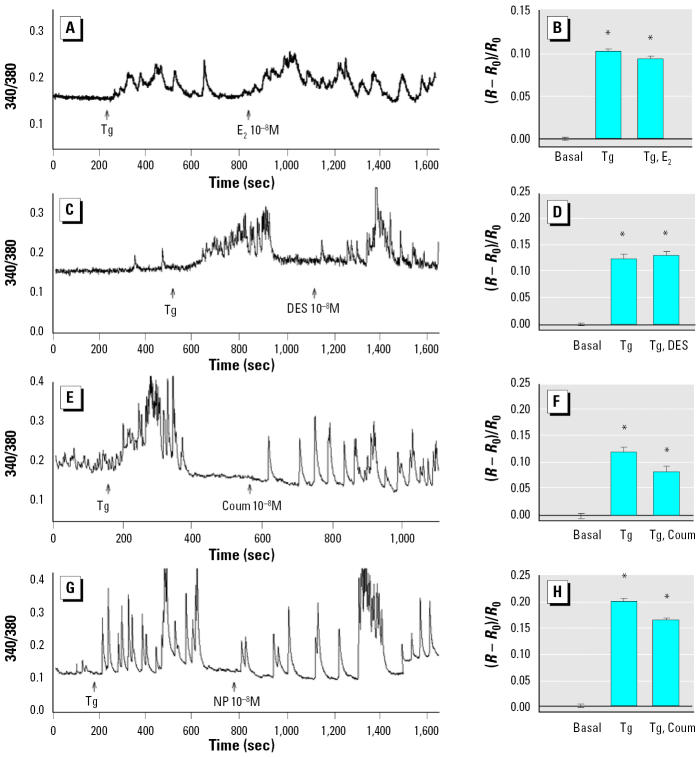
Effect of thapsigargin (Tg) on XE-induced intracellular Ca^2+^ changes. Intracellular Ca^2+^ stores were emptied by the addition of 1 μM Tg, and cells were then treated with 10^−8^ M E_2_ or XE. (*A* and *B*; *n* = 12 cells/3 experiments), DES (*C* and *D*; *n* = 12 cells/3 experiments), coumestrol (Coum; *E* and *F*; *n* = 6 cells/ 3 experiments), or *p*-nonylphenol (NP; *G* and *H*; *n* = 8 cells/3 experiments). Each trace (*A,C,E,G)* represents a single representative cell; bar graphs (*B,D,F,H*) represent multiple imaged cells over several experiments (mean ± SE).
*Statistically different from basal level (*p* < 0.05).

To determine which sources of Ca^2+^ (intracellular, extracellular, or both) were involved in the XE-induced response, we administered thapsigargin, a cell-permeable inhibitor that releases Ca^2+^ from intracellular stores by specifically and irreversibly inhibiting endoplasmic reticular Ca^2+^ ATPase (Figure 4). We first completely emptied the intracellular stores of Ca^2+^ with the application of 1 μM thapsigargin; this is evident from the rise in intracellular Ca^2+^ levels that occurred immediately after thapsigargin application. Then, after thapsigargin treatment, E_2_ and each XE could still trigger an intracellular Ca^2+^ rise, suggesting that the intracellular induced Ca^2+^ increase comes from the extracellular pool.

### Intracellular Ca^2+^ changes are due to an influx of extracellular Ca^2+^.

**Figure 5:**
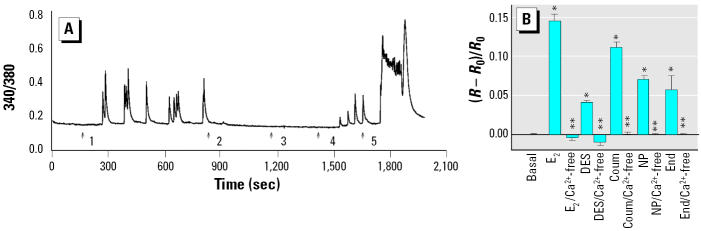
Effect of Ca^2+^-free extracellular solution on XE-induced Ca^2+^ fluxes. (*A*) Representative trace for E_2_ with the following sequential treatments: 1) 10 nM E_2_ in normal Ringer’s, 2) Ca^2+^-free Ringer’s added, 3) 10 nM E_2_ in the presence of Ca^2+^-free Ringer’s, 4) wash with normal Ringer’s, and 5) Ringer’s–20 mM KCl. (*B*) Represents the same treatment sequence shown in (*A*) for DES (*n* = 7 cells/3 experiments); coumestrol (Coum; *n* = 8 cells/3 experiments); *p*-nonylphenol (NP; *n* = 5 cells/3 experiments); and endosulfan (End; *n* = 6 cells/3 experiments). Values shown are mean ± SE for [(*R* – *R*_0_)/*R*_0_] for multiple imaged cells over several experiments.
*Statistically different from the basal level (*p* < 0.05). **Statistically different from the E_2_- or XE-treated value (*p* < 0.05).

To confirm that the Ca^2+^ increase was drawn from an extra-cellular source, we tested the effect of the presence or absence of Ca^2+^ in the solution surrounding the cells. We first triggered an intracellular Ca^2+^ change with the administration of E_2_ or XEs at 10^−8^ M in normal Ringer’s. We then eliminated Ca^2+^ from the extracellular solution by administering Ca^2+^-free Ringer’s into the perfusion system. The cells were then treated with either 10^−8^ M E_2_ or XE diluted in Ca^2+^-free Ringer’s. The effect on the response to E_2_ is shown in Figure 5A, where intracellular Ca^2+^ level increases were abolished, confirming that extracellular Ca^2+^ was the source for the intracellular Ca^2+^ elevations. These experiments were repeated for DES, coumestrol, nonylphenol, and endosulfan, with similar results (Figure 5B, averaged responses). To determine continued cell viability after treatment with Ca^2+^-free Ringer’s and estrogenic compounds, we washed out the Ca^2+^-free Ringer’s with normal Ringer’s followed by Ringer’s containing 20 mM KCl (shown only for E_2_ in Figure 5A). The cells always responded to both the normal Ringer’s (by returning of the Ca^2+^ influx pattern to the normal basal level) and the Ringer’s–20 mM KCl (by displaying a large and transient Ca^2+^ influx due to cell depolarization), thus demonstrating cell viability.

### XE-induced Ca^2+^ influx is mediated by L-type Ca^2+^ channels.

**Figure 6:**
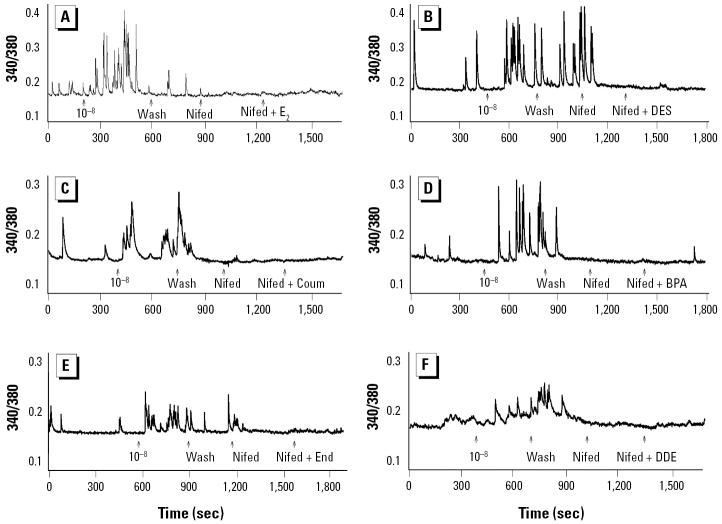
Effect of nifedipine (Nifed) on intracellular Ca^2+^ changes induced by (*A*) E_2_, (*B*) DES, (*C*) coumestrol (Coum), (*D*) BPA, (*E*) endosulfan (End), or (*F*) DDE. GH3/B6/F10 cells were treated with E_2_ or the indicated XE at 10^−8^ M, and 10 μM Nifed was then added, followed by a second addition of 10^−8^ M E_2_ or XE in the presence of 10 μM Nifed. Each trace is a single representative cell.

**Figure 7:**
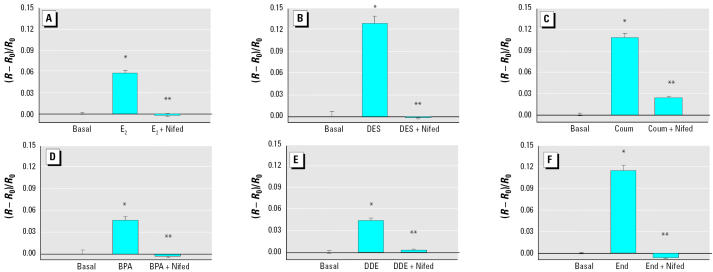
Cell effects of nifedipine (Nifed) on XE-induced intracellular Ca^2+^ increases (mean ± SE) induced by (*A*) E_2_ (*n* = 8 cells/3 experiments), (*B*) DES (*n* = 8 cells/ 3 experiments), (*C*) coumestrol (Coum; *n* = 9 cells/3 experiments), (*D*) BPA (*n* = 16 cells/4 experiments), (*E*) DDE (*n* = 9 cells/3 experiments), and (*F*) endosulfan (End; *n* = 9 cells/3 experiments). GH3/B6/F10 cells were treated with E_2_ or the indicated XE at 10^−8^ M, and 10 μM Nifed was then added, followed by a second addition of E_2_ or XE (10^−8^ M) in the presence of 10 μM Nifed. Bars indicate the change in fluorescence ratio divided by the basal fluorescence [(*R* – *R*_0_)/*R*_0_], averaged for multiple imaged cells over several experiments.
*Statistically different from the basal level (*p* < 0.05). **Statistically different from the E_2_- or XE-stimulated value (*p* < 0.05).

We have recently shown that E_2_ causes an intracellular Ca^2+^ change via the L-type Ca^2+^ channel (Bulayeva et al. 2005). XEs mimic the response caused by E_2_, implying that they may also act via the L-type Ca^2+^ channel. To test this hypothesis, we administered nifedipine, an L-type Ca^2+^ channel blocker that inhibits Ca^2+^ influx into the cell from extracellular sources. We monitored single cells (Figure 6) and then averaged these responses from multiple cells (Figure 7) to quantitate the responses. We first triggered a Ca^2+^ response by stimulating cells with 10^−8^ M E_2_ or XEs representative of different classes (DES, coumestrol, BPA, DDE, and endosulfan). After washout of the estrogens, the addition of 10 μM nifedipine caused cessation of Ca^2+^ influx. Subsequent addition of 10^−8^ M E_2_ or XE, in the presence of nifedipine, could not elicit a Ca^2+^ influx.

### XEs stimulate the rapid secretion of PRL.

**Figure 8:**
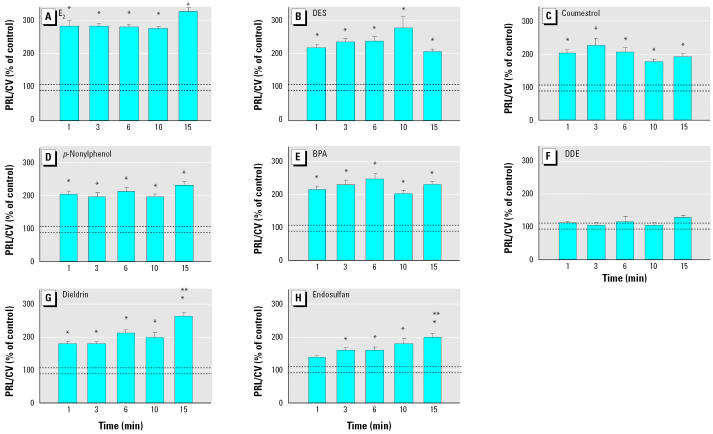
Time dependence (mean ± SE) of XE-induced PRL release in GH3/B6/F10 cells. (*A*) E_2_ (*n* = 16 samples/4 experiments), (*B*) DES (*n* = 20 samples/5 experiments), (*C*) coumestrol (*n* = 24 samples/6 experiments), (*D*) *p*-nonylphenol (*n* = 20 samples/4 experiments), (*E*) BPA (*n* = 20 samples/4 experiments), (*F*) DDE (*n* = 20 samples/ 4 experiments), (*G*) dieldrin (*n* = 20 samples/4 experiments), or (*H*) endosulfan (*n* = 20 samples/4 experiments). PRL/CV represents the percentage of the basal PRL secretion at each individual time point, normalized to the cell number [crystal violet (CV) signal]. Dashed lines indicate the error range surrounding the basal level.
*Statistically different from the basal level (*p* < 0.05). **Statistically different from the 1-min treated value (*p* < 0.05).

At 10^−8^ M E_2_ or XE, PRL was secreted rapidly (by 1 min), a response largely complete by 1–3 min (Figure 8) for most of the compounds (E_2_, DES, coumestrol, nonylphenol, and BPA). However, the organochlorine pesticides produced either delayed or no PRL secretion; DDE did not cause PRL secretion at any time point, and dieldrin and endosulfan stimulated PRL secretion incrementally over time with maximal secretion at 15 min, which was significantly different from their secretion level at 1 min.

### XEs stimulate the rapid secretion of PRL in a dose-dependent manner.

**Figure 9:**
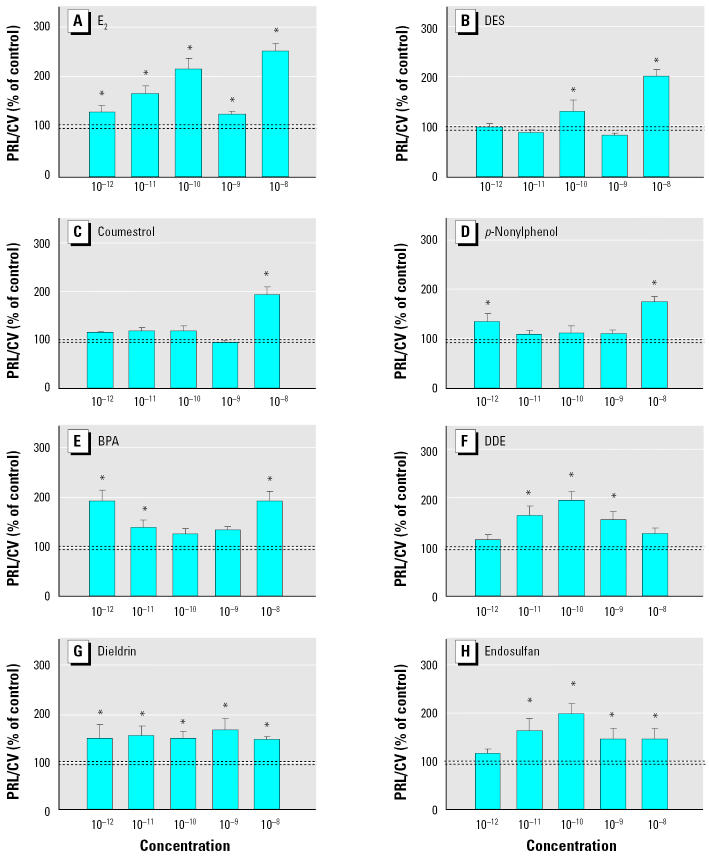
XE-induced concentration-dependent PRL release (mean ± SE) in GH3/B6/F10 cells treated for 3 min with (*A*) E_2_ (*n* = 28 samples/7 experiments), (*B*) DES (*n* = 12 samples/3 experiments), (*C*) coumestrol (*n* = 20 samples/5 experiments), (*D*) *p*-nonylphenol (*n* = 12 samples/3 experiments), (*E*) BPA (*n* = 16 samples/ 4 experiments), (*F*) DDE (*n* = 16 samples/4 experiments), (*G*) dieldrin (*n* = 20 samples/5 experiments), (*H*) endosulfan (*n* = 24 samples/6 experiments). PRL/CV represents the percentage of the basal PRL secretion at each individual time point, normalized to the cell number [crystal violet (CV) signal]. Dashed lines indicate the error range around the basal level.
*Statistically different from the basal level (*p* < 0.05).

The E_2_ or XE concentration dependence of PRL secretion at 3 min shows differences among compounds (Figure 9). The dose–response pattern for DES resembles that for E_2_, although DES is less potent at the lower concentrations. The E_2_ and DES dose–response curves were interrupted by a single lower/inactive nanomolar dose that sits between two active doses of 10^−10^ M and 10^−8^ M. Coumestrol triggered PRL release only at the highest concentration (10^−8^ M). Nonylphenol and BPA, both detergents, show a bimodal response curve with a wide gap (interruption) at the middle concentrations; PRL secretion was only elicited at the highest and lowest concentrations. The pesticides DDE and endosulfan show similar response curves, with maximal secretion at 10^−10^ M. Dieldrin induced PRL secretion at all concentrations from 10^−12^ M to 10^−8^ M, but with no apparent dose-dependent changes across these concentrations.

### PRL secretion is blocked by nifedipine.

**Figure 10:**
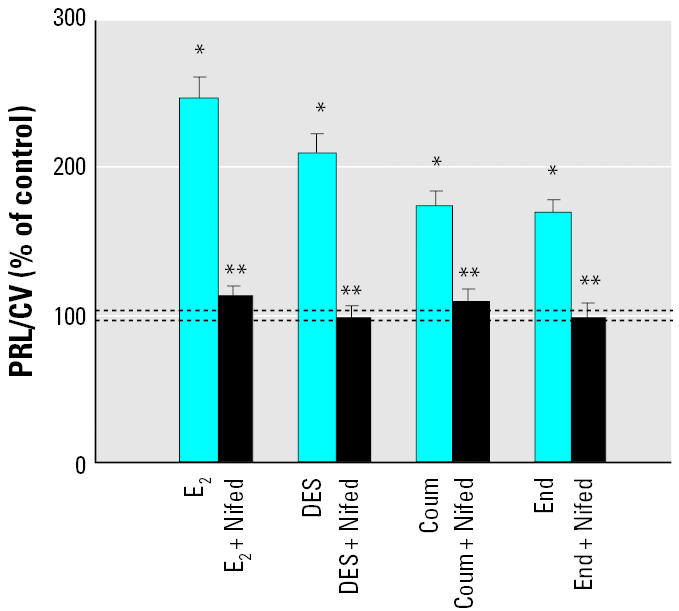
Inhibition of XE-induced PRL secretion (mean ± SE) by nifedipine (Nifed) in GH3/B6/F10 cells treated with E_2_ or XE at 10^−8^ M with or without 10 μM Nifed. E_2_ (*n* = 28 samples/7 experiments); E_2_/Nifed (*n* = 32 samples/8 experiments); DES (*n* = 24 samples/ 6 experiments); DES/Nifed (*n* = 28 samples/7 experiments); coumestrol (Coum; *n* = 24 samples/6 experiments); Coum/Nifed (*n* = 28 samples/7 experiments); endosulfan (End; *n* = 24 samples/6 experiments); End/Nifed; *n* = 24 samples/6 experiments). PRL/CV represents the percentage of the basal PRL secretion at each individual time point, normalized to the cell number [crystal violet (CV) signal]. Dashed lines indicate the error range around the basal level.
*Statistically different from the basal level (*p* < 0.05). **Statistically different from the E_2_- or XE-treated value (*p* < 0.05).

To test whether PRL secretion occurs via the L-type Ca^2+^ channel mechanism, we administered nifedipine to block the influx of Ca^2+^ (Figure 10). E_2_ and a representative set of XEs (DES, coumestrol, and endosulfan) were tested at a 10^−8^ M concentration with and without nifedipine. Nifedipine, which blocked the entry of Ca^2+^ from the extracellular solution, also abolished the E_2_- and XE-induced PRL secretion at 3 min.

## Discussion

There has been much debate about the mechanisms by which XEs act. The concentrations at which XEs are believed to be mechanistically active have largely been determined by assaying for the transcriptional activity of these compounds via the well-established nuclear pathway of action for steroids. We hypothesized that XEs, like steroid hormones, can elicit both delayed (genomic) and rapid (nongenomic or membrane-initiated) responses (Bulayeva et al. 2004; Bulayeva and Watson 2004; Watson and Gametchu 2003). We established a cell model with which to screen the rapid non-genomic activities of estrogenic compounds using cells naturally expressing high levels of a membrane form of ER-αthought to mediate these nongenomic actions. We previously demonstrated that these cells can respond rapidly to E_2_ by extracellular signal-regulated kinase (ERK) phosphorylation (Bulayeva et al. 2004; Bulayeva and Watson 2004) and PRL release (Norfleet et al. 2000; Pappas et al. 1994). We also recently determined that E_2_ induces a rapid Ca^2+^ influx (within 30 sec) via the L-type Ca^2+^ channel in these cells, which is necessary for rapidly induced PRL secretion (Bulayeva et al. 2005). However, the ability of XEs to induce mechanistic pathways related to secretion of hormones associated with endocrine-disruptive mechanisms has largely not been determined. In the present studies, we demonstrated the ability of very low (picomolar to nanomolar) concentrations of several XEs to induce a rapid Ca^2+^ influx resulting in PRL secretion.

Because we found that some XEs induced rapid mitogen-activated protein kinase (MAPK) activation via the same membrane-initiated signaling pathway used by E_2_ in pituitary tumor cells (Bulayeva and Watson 2004), we hypothesized that XEs could mimic E_2_ in other rapid signal-generating mechanisms, including effects on intracellular Ca^2+^ levels. Our present studies thus show that, like E_2_ (Bulayeva et al. 2005), XEs potently induce intracellular Ca^2+^ increases in our mER-α–enriched rat prolactinoma cell line GH3/B6/F10, whereas mER-α–deficient cells cannot respond. Both physiological estrogen (E_2_) and XEs stimulate a rapid Ca^2+^ influx (within 30 sec) from the extracellular media (because elimination of extracellular Ca^2+^ abolished intracellular Ca^2+^ changes) that is independent of the release of endoplasmic reticulum Ca^2+^ stores (i.e., is thapsigargin insensitive). Blocking the L-type Ca^2+^ channels with nifedipine also abrogated XE-induced Ca^2+^ influx. Therefore, the XEs used in our study were able to cause E_2_-like changes in Ca^2+^ levels via similar mechanisms.

Increases in Ca^2+^ levels often lead to the release of many different kinds of stored hormones and other proteins from secretory vesicles (Kits and Mansvelder 2000). GH3/B6 cells manufacture and spontaneously secrete PRL (Zyzek et al. 1981); however, in addition to constitutive PRL secretion, a variety of external stimuli such as drugs or hormones can enhance the release of stored PRL from vesicles [e.g., E_2_-induced PRL release (Pappas et al. 1994)]. These data demonstrate that environmental contaminants such as XEs of different classes (plastics manufacturing and detergent byproducts, pesticides, phytoestrogens, and synthetic estrogens) can mimic endogenous estrogens such as E_2_, causing rapid PRL secretion by raising cellular Ca^2+^ levels.

PRL is conventionally viewed as a pituitary hormone that stimulates and maintains the secretion of milk. However, PRL is also synthesized and secreted by a broad range of cells, including those of the immune system (DiMattia et al. 1988; Pellegrini et al. 1992), breast cancers (Clevenger et al. 1995), and the lining of the pregnant uterus (Gellersen et al. 1991; Zetser et al. 2001). Physiological stimuli such as increased levels of ovarian steroids (primarily estrogens) can increase PRL secretion, leading to delay in puberty (Barrio et al. 1979), interference with ovulation (Bole-Feysot et al. 1998; McNeilly et al. 1982), decreases in libido and fertility (Gurbuz et al. 2003; Heller and Jacobs 1978; Sodersten et al. 1983), and cell proliferation (Krown et al. 1992; Sauro and Zorn 1991). Behavioral effects of PRL are also known (Bridges et al. 1985; Lucas et al. 1998). Therefore, overstimulation, inappropriate stimulation (for developmental stage or reproductive cycle stage), or inhibition of PRL secretion can lead to a variety of disruptions of normal reproductive function, and our data demonstrate that XEs at low concentrations could cause such altered PRL secretion.

Each XE that we studied produced PRL release during the first 15 min of application, but most elicited a significant response by 1 min. Dose–response curves revealed intermediate inactive doses, as we (Bulayeva and Watson 2004; Watson et al. 1999b) and others (Picotto et al. 1996) have previously observed, although we still do not have a substantiated explanation for such response gaps. Receptors that activate such responses via the membrane may be sequestered on different cellular surfaces or in different membrane compartments such as rafts and caveolae (Razandi et al. 2002; Shaul 2002), which could produce different receptor subpopulations with different dose–response characteristics. It is interesting to note that the Ca^2+^ response did not display such a bimodal dose–response pattern, so additional signaling mechanisms besides Ca^2+^ must be responsible for these differential dose patterns, as we have suggested previously in studies that noted differences between E_2_- and KCl- induced Ca^2+^ levels and resulting PRL secretion (Bulayeva et al. 2005).

Increased Ca^2+^ levels can trigger the release of PRL and other hormones from secretory vesicles, but it can also initiate signaling cascades leading to a variety of kinase activations (e.g., adenylyl cyclase production of cAMP leading to activation of protein kinase A, phospholipase C activation resulting in activation of protein kinase C, calmodulin activation of pathways leading to MAP kinase phosphorylation, etc.), resulting in changes in the phosphorylation status of a variety of cellular proteins leading to rapid functional consequences. These signaling cascades are now known to be rapidly stimulated by steroid hormones (reviewed by Watson and Gametchu 1999, 2003). For example, E_2_ induces a rapid increase in cAMP that parallels the changes in Ca^2+^ uptake in duodenal cells (Picotto et al. 1996), and E_2_ has been shown to increase cytosolic Ca^2+^ levels as well as induce MAPK activation in hemocytes (Canesi et al. 2004). Steroid-induced Ca^2+^ influxes have also been reported in ovarian, prostate, cardiac and vascular smooth muscle, and bone cells (reviewed by Watson and Gametchu 1999). The list of cell types and stages in which steroids can induce Ca^2+^ changes is rapidly growing and becoming a hallmark of non-genomic steroid action; examples now exist for most classes of steroids (reviewed by Watson 2003; Watson and Gametchu 1999, 2003).

Some researchers have speculated that the ER that mediates XE effects is a unique receptor (Ghosh et al. 1999; Nadal et al. 2000). However, our studies demonstrate that sub-clones of GH3/B6 cells that substantially lack the membrane version of ER-α[D9 subline (Pappas et al. 1994)] cannot respond to E_2_ or XEs via Ca^2+^ fluxes, PRL release (these studies), or ERK activation (Bulayeva et al. 2004; Bulayeva and Watson 2004). This is in keeping with our previous investigations that indicated the involvement of an ER-αprotein in these rapid responses in several ways. E_2_-induced PRL release was blocked in cells inhibited with the ER antagonist ICI 182,870 (Bulayeva et al. 2005) or treated with specific ER-αantibodies (Norfleet et al. 2000). The mER expression in these same cells was abolished by ER-αby antisense strategies (Norfleet et al. 1999). Of course, it is possible that other components besides mER-αare necessary, but the lack of ER-αin the membrane prevents signaling responses to these compounds.

XEs pose a potential environmental threat to human health because experimental animal exposures have demonstrated endocrine developmental anomalies at levels similar to those sometimes seen in environmental contamination. However, most previous studies have emphasized the genomic mechanisms of XE action, which require very high concentrations. Our research has instead focused on the rapid, nongenomic or membrane-initiated effects of these environmental contaminants. Via these alternate signaling pathways, estrogen mimetics such as XEs could interfere with endogenous estrogen actions via multiple mechanisms. By eliciting, enhancing, or inhibiting estrogenic signaling, they may interfere with physiological estrogenic signals, affecting many downstream functions. Each XE however, shows unique temporal and dose-responsive patterns, possibly due to the differential involvement of companion signaling pathways. Although we have linked XEs to rapid cellular events that trigger intracellular Ca^2+^ influxes and PRL secretion, further study is needed to fully understand all of these differentially activated signaling cascades and their relationships to the myriad outcomes of XE exposure.

## References

[b1-ehp0113-000431] Barrio R, Roger M, Chaussain JL, Job JC (1979). Prolactin-secreting pituitary adenomas in children and adolescents. Study of a series of 8 cases [in French]. Arch Fr Pediatr.

[b2-ehp0113-000431] Bole-Feysot C, Goffin V, Edery M, Binart N, Kelly PA (1998). Prolactin (PRL) and its receptor: actions, signal transduction pathways and phenotypes observed in PRL receptor knockout mice. Endocr Rev.

[b3-ehp0113-000431] Bridges RS, DiBiase R, Loundes DD, Doherty PC (1985). Prolactin stimulation of maternal behavior in female rats. Science.

[b4-ehp0113-000431] Brown NM, Lamartiniere CA (1995). Xenoestrogens alter mammary gland differentiation and cell proliferation in the rat. Environ Health Perspect.

[b5-ehp0113-000431] Bulayeva NN, Gametchu B, Watson CS (2004). Quantitative measurement of estrogen-induced ERK 1 and 2 activation via multiple membrane-initiated signaling pathways. Steroids.

[b6-ehp0113-000431] Bulayeva NN, Watson CS (2004). Xenoestrogen-induced ERK 1 and 2 activation via multiple membrane-initiated signaling pathways. Environ Health Perspect.

[b7-ehp0113-000431] Bulayeva NN Wozniak AL Lash LL Watson CS 2005 Mechanisms of membrane estrogen receptor-α-mediated rapid stimulation of Ca2+ levels and prolactin release in a pituitary cell line Am J Physiol Endocrinol Metab 288 E388 E39710.1152/ajpendo.00349.2004 [online 19 October 2004]. 15494610 10.1152/ajpendo.00349.2004

[b8-ehp0113-000431] Campbell AK (1990). Calcium as an intracellular regulator. Proc Nutr Soc.

[b9-ehp0113-000431] Canesi L, Ciacci C, Betti M, Lorusso LC, Marchi B, Burattini S (2004). Rapid effects of 17β-estradiol on cell signaling and function of *Mytilus* hemocytes. Gen Comp Endocrinol.

[b10-ehp0113-000431] Clevenger CV, Chang WP, Ngo W, Pasha TL, Montone KT, Tomaszewski JE (1995). Expression of prolactin and prolactin receptor in human breast carcinoma. Evidence for an autocrine/paracrine loop. Am J Pathol.

[b11-ehp0113-000431] Derfoul A, Lin FJ, Awumey EM, Kolodzeski T, Hall DJ, Tuan RS (2003). Estrogenic endocrine disruptive components interfere with calcium handling and differentiation of human trophoblast cells. J Cell Biochem.

[b12-ehp0113-000431] DiMattia GE, Gellersen B, Bohnet HG, Friesen HG (1988). A human B-lymphoblastoid cell line produces prolactin. Endocrinology.

[b13-ehp0113-000431] Freeman ME, Kanyicska B, Lerant A, Nagy G (2000). Prolactin: structure, function, and regulation of secretion. Physiol Rev.

[b14-ehp0113-000431] Gellersen B, Bonhoff A, Hunt N, Bohnet HG (1991). Decidual-type prolactin expression by the human myometrium. Endocrinology.

[b15-ehp0113-000431] Ghosh D, Taylor JA, Green JA, Lubahn DB (1999). Methoxychlor stimulates estrogen-responsive messenger ribonucleic acids in mouse uterus through a non-estrogen receptor (non-ER) alpha and non-ER beta mechanism. Endocrinology.

[b16-ehp0113-000431] Gotz F, Thieme S, Dorner G (2001). Female infertility—effect of perinatal xenoestrogen exposure on reproductive functions in animals and humans. Folia Histochem Cytobiol.

[b17-ehp0113-000431] Gray LE, Wolf C, Lambright C, Mann P, Price M, Cooper RL (1999). Administration of potentially antiandrogenic pesticides (procymidone, linuron, iprodione, chlozolinate, *p*,*p*′-DDE, and ketoconazole) and toxic substances (dibutyl-and diethylhexyl phthalate, PCB 169, and ethane dimethane sulphonate) during sexual differentiation produces diverse profiles of reproductive malformations in the male rat. Toxicol Ind Health.

[b18-ehp0113-000431] Gurbuz B, Yalti S, Ficicioglu C, Ozden S, Yildirim G, Sayar C (2003). Basal hormone levels in women with recurrent pregnancy loss. Gynecol Endocrinol.

[b19-ehp0113-000431] Hatch EE, Herbst AL, Hoover RN, Noller KL, Adam E, Kaufman RH (2001). Incidence of squamous neoplasia of the cervix and vagina in women exposed prenatally to diethylstilbestrol (United States). Cancer Causes Control.

[b20-ehp0113-000431] Heller ME, Jacobs HS (1978). Prolactin and infertility. Fertil Contracept.

[b21-ehp0113-000431] Huang JK, Jan CR (2001). Mechanism of estrogens-induced increases in intracellular Ca(2+) in PC3 human prostate cancer cells. Prostate.

[b22-ehp0113-000431] Kits KS, Mansvelder HD (2000). Regulation of exocytosis in neuro-endocrine cells: spatial organization of channels and vesicles, stimulus-secretion coupling, calcium buffers and modulation. Brain Res Brain Res Rev.

[b23-ehp0113-000431] Kloas W, Lutz I, Einspanier R (1999). Amphibians as a model to study endocrine disruptors: II. Estrogenic activity of environmental chemicals in vitro and in vivo. Sci Total Environ.

[b24-ehp0113-000431] Krown KA, Wang YF, Ho TW, Kelly PA, Walker AM (1992). Prolactin isoform 2 as an autocrine growth factor for GH3 cells. Endocrinology.

[b25-ehp0113-000431] Laessig SA, McCarthy MM, Silbergeld EK (1999). Neurotoxic effects of endocrine disruptors. Curr Opin Neurol.

[b26-ehp0113-000431] Lee PC (1998). Disruption of male reproductive tract development by administration of the xenoestrogen, nonylphenol, to male newborn rats. Endocrine.

[b27-ehp0113-000431] Loomis AK, Thomas P (2000). Effects of estrogens and xeno-estrogens on androgen production by Atlantic croaker testes in vitro: evidence for a nongenomic action mediated by an estrogen membrane receptor. Biol Reprod.

[b28-ehp0113-000431] Lucas BK, Ormandy CJ, Binart N, Bridges RS, Kelly PA (1998). Null mutation of the prolactin receptor gene produces a defect in maternal behavior. Endocrinology.

[b29-ehp0113-000431] Massaad C, Barouki R (1999). An assay for the detection of xenoestrogens based on a promoter containing overlapping EREs. Environ Health Perspect.

[b30-ehp0113-000431] Mathur V, Bhatnagar P, Sharma RG, Acharya V, Sexana R (2002). Breast cancer incidence and exposure to pesticides among women originating from Jaipur. Environ Int.

[b31-ehp0113-000431] McNeilly AS, Glasier A, Jonassen J, Howie PW (1982). Evidence for direct inhibition of ovarian function by prolactin. J Reprod Fertil.

[b32-ehp0113-000431] Nadal A, Ropero AB, Laribi O, Maillet M, Fuentes E, Soria B (2000). Nongenomic actions of estrogens and xenoestrogens by binding at a plasma membrane receptor unrelated to estrogen receptor alpha and estrogen receptor beta. Proc Natl Acad Sci USA.

[b33-ehp0113-000431] Norfleet AM, Clarke C, Gametchu B, Watson CS (2000). Antibodies to the estrogen receptor-αmodulate prolactin release from rat pituitary tumor cells through plasma membrane estrogen receptors. FASEB J.

[b34-ehp0113-000431] Norfleet AM, Thomas ML, Gametchu B, Watson CS (1999). Estrogen receptor-αdetected on the plasma membrane of aldehyde-fixed GH3/B6/F10 rat pituitary cells by enzyme-linked immunocytochemistry. Endocrinology.

[b35-ehp0113-000431] Oka T, Adati N, Shinkai T, Sakuma K, Nishimura T, Kurose K (2003). Bisphenol A induces apoptosis in central neural cells during early development of *Xenopus laevis*. Biochem Biophys Res Commun.

[b36-ehp0113-000431] Palmer JR, Hatch EE, Rao RS, Kaufman RH, Herbst AL, Noller KL (2001). Infertility among women exposed prenatally to diethylstilbestrol. Am J Epidemiol.

[b37-ehp0113-000431] Pappas TC, Gametchu B, Yannariello-Brown J, Collins TJ, Watson CS (1994). Membrane estrogen receptors in GH3/B6 cells are associated with rapid estrogen-induced release of prolactin. Endocrine.

[b38-ehp0113-000431] Pellegrini I, Lebrun JJ, Ali S, Kelly PA (1992). Expression of prolactin and its receptor in human lymphoid cells. Mol Endocrinol.

[b39-ehp0113-000431] Picotto G, Massheimer V, Boland R (1996). Acute stimulation of intestinal cell calcium influx induced by 17 beta-estradiol via the cAMP messenger system. Mol Cell Endocrinol.

[b40-ehp0113-000431] Razandi M, Oh P, Pedram A, Schnitzer J, Levin ER (2002). ERs associate with and regulate the production of caveolin: implications for signaling and cellular actions. Mol Endocrinol.

[b41-ehp0113-000431] Sauro MD, Zorn NE (1991). Prolactin induces proliferation of vascular smooth muscle cells through a protein kinase C-dependent mechanism. J Cell Physiol.

[b42-ehp0113-000431] Shaul PW (2002). Regulation of endothelial nitric oxide synthase: location, location, location. Annu Rev Physiol.

[b43-ehp0113-000431] Singleton DW, Khan SA (2003). Xenoestrogen exposure and mechanisms of endocrine disruption. Front Biosci.

[b44-ehp0113-000431] Sodersten P, Hansen S, Eneroth P (1983). Inhibition of sexual behaviour in lactating rats. J Endocrinol.

[b45-ehp0113-000431] Sonnenschein C, Soto AM (1998). An updated review of environmental estrogen and androgen mimics and antagonists. J Steroid Biochem Mol Biol.

[b46-ehp0113-000431] Soto AM, Sonnenschein C, Chung KL, Fernandez MF, Olea N, Serrano FO (1995). The E-SCREEN assay as a tool to identify estrogens: an update on estrogenic environmental pollutants. Environ Health Perspect.

[b47-ehp0113-000431] Steinmetz R, Mitchner NA, Grant A, Allen DL, Bigsby RM, Ben-Jonathan N (1998). The xenoestrogen bisphenol A induces growth, differentiation, and c-*fos* gene expression in the female reproductive tract. Endocrinology.

[b48-ehp0113-000431] Stevens JT, Breckenridge CB, Wetzel LT, Gillis JH, Luempert LG, Eldridge JC (1994). Hypothesis for mammary tumori-genesis in Sprague-Dawley rats exposed to certain triazine herbicides. J Toxicol Environ Health.

[b49-ehp0113-000431] Sumpter JP (1995). Feminized responses in fish to environmental estrogens. Toxicol Lett.

[b50-ehp0113-000431] vom Saal FS, Timms BG, Montano MM, Palanza P, Thayer KA, Nagel SC (1997). Prostate enlargement in mice due to fetal exposure to low doses of estradiol or diethylstilbestrol and opposite effects at high doses. Proc Natl Acad Sci USA.

[b51-ehp0113-000431] Watson CS ed. 2003. The Identities of Membrane Steroid Receptors … and Other Proteins Mediating Nongenomic Steroid Action. Boston:Kluwer Academic Publishers.

[b52-ehp0113-000431] Watson CS, Campbell CH, Gametchu B (1999a). Membrane estrogen receptors on rat pituitary tumor cells: immuno-identification and responses to estradiol and xenoestrogens. Exp Physiol.

[b53-ehp0113-000431] Watson CS, Gametchu B (1999). Membrane-initiated steroid actions and the proteins that mediate them. Proc Soc Exp Biol Med.

[b54-ehp0113-000431] Watson CS, Gametchu B (2003). Proteins of multiple classes participate in nongenomic steroid actions. Exp Biol Med.

[b55-ehp0113-000431] Watson CS, Norfleet AM, Pappas TC, Gametchu B (1999b). Rapid actions of estrogens in GH_3_/B6 pituitiary tumor cells via a plasma membrane version of estrogen receptor-α. Steroids.

[b56-ehp0113-000431] Witorsch RJ (2002). Endocrine disruptors: can biological effects and environmental risks be predicted?. Regul Toxicol Pharmacol.

[b57-ehp0113-000431] Zetser A, Kisliouk T, Ivakin E, Lahav M (2001). Dependence on prolactin of the luteolytic effect of prostaglandin F2alpha in rat luteal cell cultures. Biol Reprod.

[b58-ehp0113-000431] Zyzek E, Dufy-Barbe L, Dufy B, Vincent JD (1981). Short-term effect of estrogen on release of prolactin by pituitary cells in culture. Biochem Biophys Res Commun.

